# Merlin regulates signaling events at the nexus of development and cancer

**DOI:** 10.1186/s12964-020-00544-7

**Published:** 2020-04-16

**Authors:** Mateus Mota, Lalita A. Shevde

**Affiliations:** 1 Department of Pathology, University of Alabama at Birmingham, WTI 320D, 1824 6th Avenue South, Birmingham, AL 35233 USA; 2 O’Neal Comprehensive Cancer Center, University of Alabama at Birmingham, WTI 320D, 1824 6th Avenue South, Birmingham, AL 35233 USA

**Keywords:** Merlin, Development, Wnt, Hedgehog, Notch, Hippo, TGF-beta, Cancer

## Abstract

**Background:**

In this review, we describe how the cytoskeletal protein Merlin, encoded by the Neurofibromin 2 (*NF2*) gene, orchestrates developmental signaling to ensure normal ontogeny, and we discuss how Merlin deficiency leads to aberrant activation of developmental pathways that enable tumor development and malignant progression.

**Main body:**

Parallels between embryonic development and cancer have underscored the activation of developmental signaling pathways. Hippo, WNT/β-catenin, TGF-β, receptor tyrosine kinase (RTK), Notch, and Hedgehog pathways are key players in normal developmental biology. Unrestrained activity or loss of activity of these pathways causes adverse effects in developing tissues manifesting as developmental syndromes. Interestingly, these detrimental events also impact differentiated and functional tissues. By promoting cell proliferation, migration, and stem-cell like phenotypes, deregulated activity of these pathways promotes carcinogenesis and cancer progression. The *NF2* gene product, Merlin, is a tumor suppressor classically known for its ability to induce contact-dependent growth inhibition. Merlin plays a role in different stages of an organism development, ranging from embryonic to mature states. While homozygous deletion of *Nf2* in murine embryos causes embryonic lethality, Merlin loss in adult tissue is implicated in Neurofibromatosis type 2 disorder and cancer. These manifestations, cumulatively, are reminiscent of dysregulated developmental signaling.

**Conclusion:**

Understanding the molecular and cellular repercussions of Merlin loss provides fundamental insights into the etiology of developmental disorders and cancer and has the potential, in the long term, to identify new therapeutic strategies.

**Video Abstract**

**Graphical abstract:**

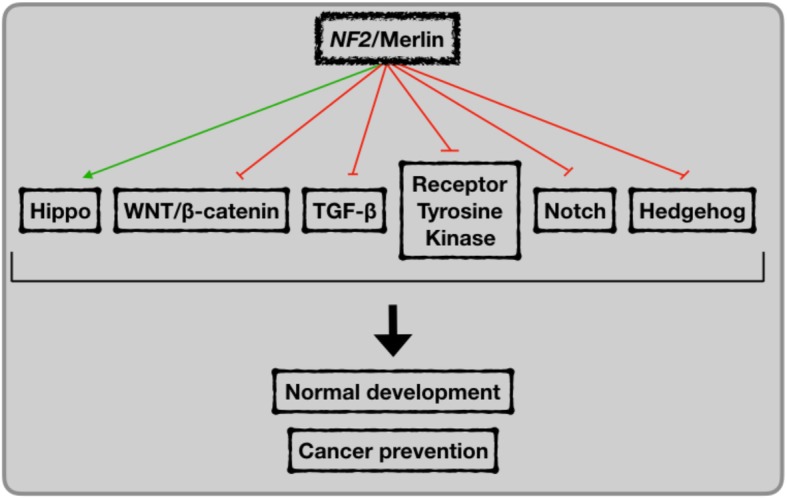

## Background

The human Neurofibromin 2 (*NF2)* gene is located on chromosome 22, contains 17 exons, and directs the synthesis of a 70-kDa protein named NF2 or Merlin. High *NF2* expression was initially observed in the nervous system, more specifically in Schwann cells, meninges, and ependyma, and to some extent in the lens and nerves as well. Merlin regulates cell behavior by integrating extracellular cues with intracellular responses and acts as a classical tumor suppressor. Functionally, Merlin inhibits tumorigenesis by inducing contact-dependent growth inhibition [1].

Merlin shares more than 50% sequence homology with the Ezrin/Radixin/Moesin (ERM) family of proteins, and indeed, its nomenclature represents an acronym for “**M**oesin-**E**zrin-**R**adixin-**Li**ke Protei**n**” (Merlin). In general, the N-terminal region of cytoskeleton-associated proteins, such as the 4.1 and ERM families, contains a unique module denominated as the **4**.1, **ERM** (FERM) domain. The FERM family of proteins, including Merlin, are mainly located near the cell membrane where they bind to cytoplasmic tails of transmembrane receptors and to actin filaments through their N-terminal FERM and C-terminal domains, respectively. However, Merlin does not bind actin via its C-terminal domain; instead its interaction with actin is mediated via the FERM domain [2]. Merlin mediates contact-dependent growth inhibition in its closed form, characterized by the binding between its N-terminal FERM and C-terminal domains. This is induced by anti-mitogenic signals, such as cadherin-dependent cell-cell adhesion and CD44-dependent MYPT1 phosphatase, that keep Serine 518 (Ser518) at Merlin’s C-terminal domain dephosphorylated. However, promitogenic signals initiated by integrins and receptor tyrosine kinases activate Rho family of GTPases, such as Cdc42 and Rac, which in turn activate p21-activated kinase (PAK). PAK phosphorylates Ser518, disrupting the binding between the N-terminal FERM and C-terminal domains. In this now open structure, the tumor suppressor role of Merlin is inhibited. Merlin’s phosphorylation and inactivation are also promoted by cyclic AMP-protein kinase A (PKA) signaling and by AKT-dependent phosphorylation and ubiquitination, which cause proteasome-mediated degradation of Merlin [3].

Mutations in *NF2* causatively result in Neurofibromatosis type 2, an autosomal dominant disorder mainly associated with benign tumors in the nervous system, such as bilateral vestibular schwannomas, meningiomas, and ependymomas [2]. Although lack of functional *NF2*/Merlin activity was initially investigated in Neurofibromatosis type 2 disorder, Merlin deficiency is recognized in malignant tumors, including mesothelioma, prostate cancer, melanoma, glioma, and breast cancer [2]. The onset and progression of cell proliferative disorders are intimately connected to deregulation of cell signaling pathways that govern developmental processes. Therefore, the association of *NF2/*Merlin malfunction with both benign and malignant disease, suggests that Merlin likely regulates cell signaling associated with developmental programs. Hippo, WNT/β-catenin, TGF-β, receptor tyrosine kinase (RTK), Notch, and Hedgehog signaling pathways are the main coordinators of developmental biology [4]. In this review, we discuss how, in a spatiotemporal-dependent manner, Merlin may contribute to either activation or inhibition of developmental pathways in order to maintain cell integrity, tissue organization, and adequate overall development. Much of the current knowledge describing a possible role for Merlin in regulating components of these pathways comes from the impact of *NF2*/Merlin deficiency on the activity of these developmental biology-associated signaling events that impinge upon a malignant phenotype.

## Merlin’s role in developmental processes

Many steps of development consist in a sequence of well-coordinated events involving cell proliferation and differentiation, resulting in an organized and functional tissue. Normal embryo patterning is important before and during gastrulation, a crucial early stage of vertebrate embryogenesis, which results in the formation of the germ layers that will support organogenesis [5]. Early studies interrogating the role of *Nf2* in vivo discovered that homozygous *Nf2* mutation in murine embryos is lethal, with developmental failure occurring by embryonic days 6.5 and 7. Merlin presented as a crucial component in the organization of extraembryonic structure. The extraembryonic ectoderm of homozygous *Nf2-*mutated embryos failed to develop into an epithelial tissue, compromising their orientation and elongation. These irregular-shaped mutant embryos had impaired production of mesoderm, failed to gastrulate, and became embryonically inviable [6]. Recognizing Merlin’s role in inducing contact dependent growth inhibition, it is reasonable to infer that Merlin controls cell proliferation of the extraembryonic compartment and further contributes to an optimal gastrulation process.

Merlin’s anti-proliferative activity continues to be critical even after gastrulation phase and is crucial for the fusion of epithelial tissue, such as in the transition from neural plate to a neural tube in vertebrate embryos. For instance, a nerve cell-specific *Nf2* knockout mouse model under the control of Nestin promoter exhibited neural tube defects such as encephalocele and exencephaly [7]. Furthermore, although heterozygous *Nf2* knockout embryos are viable, the mice usually develop tumors, such as osteosarcosmas, fibrosarcoma, and hepatocellular carcinoma, with high metastatic rate to the lungs and liver [8].

Dysregulated signaling activity of pathways known for instructing embryonic development, such as Hippo, WNT/β-catenin, TGF-β, receptor tyrosine kinases, Notch, and Hedgehog usually leads to cell overproliferation and consequently lack of differentiation and functionality, resulting in abnormal embryonic formation and malignancies in developed tissues [4]. Undoubtedly, regulation of cell proliferation at either the embryonic or mature stage is imperative to the development and maintenance of an organism. Therefore, Merlin may be orchestrating these developmental signaling pathways to assure well-coordinated proliferation events, justifying its indispensable roles in preventing both failure of embryonic development and tumorigenesis.

Controlled cell proliferation is at the core of many embryonic developmental phases. For instance, proliferation of epithelial or endothelial cells into tubules, a developmental program termed branching morphogenesis, is a fundamental step in the formation of several mammalian organs, such as kidney, lung, pancreas, and mammary gland [9]. Furthermore, cell cycle progression is associated with cell polarity, a characteristic that is crucial for the establishment of an organized and functional tissue in metazoans. For instance, planar-cell polarity (PCP) is established orthogonally to apical-basal polarity and is responsible for cell behavior across the plane of the tissue. PCP defines the dorsoventral and anteroposterior body axes in vertebrates and invertebrates and is required for tissue patterning, vertebrate gastrulation, and neural tube closure [10, 11]. By controlling cell proliferation and adhesion, Merlin is instrumental for induction of cell polarity and together with the establishment of PCP, ensures epithelial tissue function in the skin and kidney for example [12, 13]. Overall, Merlin presents as a promising regulator of cell proliferation and organization through its association with developmental signaling pathways.

## Merlin and developmental signaling pathways

### Hippo signaling pathway

**Fig. 1:**
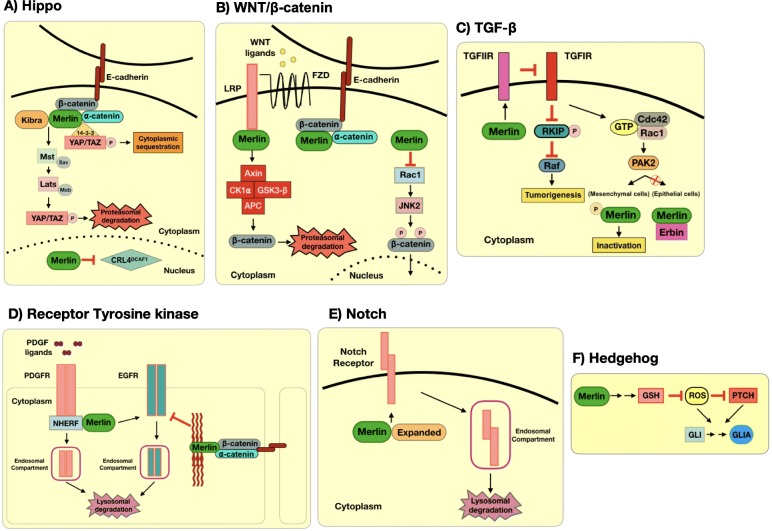
Merlin regulates different developmental signaling pathways. Merlin **a)** enables Hippo-dependent YAP/TAZ destruction, **b)** restrains β-catenin nuclear activity in the WNT pathway**, c)** regulates TGF-β signaling activation, **d)** restricts activation of PGFR and EGFR, **e)** controls Notch receptor levels availability, and **f)** disturbs the oxidative milieu resulting in the activation of Hh signaling pathway

First described as a regulator of imaginal disc growth in fruit flies (*Drosophila melanogaster)* by inducing cell-cycle arrest and apoptosis, the Hippo signaling pathway was later associated with organ growth restriction [14]. Hippo was also found to be involved in neuroepithelial cell differentiation in the optic lobe and dendrite morphogenesis in sensory neurons of *Drosophila* larvae [15]. Merlin, in cooperation with the transducer protein Kibra, is a classical activator of Hippo signaling and regulates the organization of embryonic and adult tissues/organs [3]. Merlin/Kibra serves as a scaffolding complex that localizes Hippo-associated Mst and Lats kinases to the cell membrane. Lats phosphorylates YAP/TAZ co-activators, resulting in their retention in the cytoplasm and degradation by the proteasome system. Lack of Hippo signaling results in translocation of YAP/TAZ to the nucleus and binding to TEAD transcription factors, which activate the expression of genes usually involved in tissue growth [14]. Merlin also supports Hippo activation by directly associating with ⍺-catenin and facilitating its binding to 14–3-3 protein for cytoplasmic sequestration of YAP/TAZ. Furthermore, although predominantly a cystoskeletal protein, Merlin can translocate to the nucleus, bind to DCAF1, a substrate adaptor of the CRL4-E3 ubiquitin ligase, and inhibit overall activity of CRL4^DCAF1^ ligase. By ubiquitinating histones and other chromatin-associated factors, the CRL4^DCAF1^ ligase system facilitates the activation of some YAP/TAZ targets [3]. Thus, Merlin relies on different mechanisms to tightly control YAP/TAZ transcriptional function (Fig. 1A).

Abnormal YAP/TAZ activity due to Hippo deficiency contributes to tumorigenesis, cancer progression, and metastasis with most striking cases observed in breast cancer where Hippo dysregulation is necessary for disease progression and can be detected in up to 70% of advanced cases [16]. Interestingly, Merlin loss has been correlated with increased grade in breast cancer which might justify the downregulation of Hippo signaling and increase in YAP/TAZ activity in this type of malignancy [17]. Recognizing the role of Merlin-Hippo axis in the control of tissue development and prevention of tumorigenesis, Merlin/Lats2 represses YAP/TAZ and allows the appropriate course of branching morphogenesis in murine kidney, avoiding renal hypodysplasia [18]. However, in a different context, unrestrained YAP/TAZ can induce abnormal renal cell growth, promote intratubular neoplasia and further cause clear cell renal cell carcinoma [16]. These discoveries highlight the relationship between Merlin and Hippo in maintaining tissue homeostasis and cancer.

### WNT/β-catenin signaling pathway

The WNT/β-catenin signaling pathway is crucial for embryonic developmental processes, especially in the regulation of PCP. In addition, WNT/β-catenin renews the pluripotent embryonic stem cell pool and maintains proper proliferation and differentiation in adult tissues [19, 20], such as in the plasticity of the mammary gland in development and during pregnancy and lactation. The canonical WNT/β-catenin activation is blocked by a destruction complex (Axin, adenomatous polyposis coli (APC), CKI⍺ and GSK3β) that eliminates β-catenin by proteasomal degradation. This inhibitory status is reversed when secreted WNT ligands bind to and activate Frizzled (FZD) transmembrane receptor and LRP co-receptor. The ensuing signaling events result in nuclear translocation of β-catenin and together with TCF/LEF transcription factors, culminate in activation of target gene expression [21].

Unrestrained WNT activity can cause malignant transformation and lead to development of cancers, such as colorectal, prostate, and breast tumors [22]. For instance, overexpression of WNT7A ligand and the FZD7 receptor promotes stem cell phenotype in the mammary tissue and can foster basal-like breast cancer. Furthermore, overexpression of FZD6 and FZD8 receptors exacerbates motility, invasion and metastatic potential in triple negative breast cancer cells [23]. The fact that these type of tumors also show some levels of Merlin loss or inactivation [2, 24] suggests a connection between Merlin and the WNT/β-catenin pathway. Moreover, Neurofibromatosis type 2 patients with schwannoma, a clinical manifestation of the disorder, accumulate high levels of β-catenin, further indicating that elevated WNT/ β-catenin signaling activity is associated with deficient Merlin expression [21]***.***

Indeed, it has been reported that Merlin’s N-terminal FERM domain binds to LRP6, inhibits its phosphorylation, and maintains a functional β-catenin destruction complex thereby curbing cytoplasmic β-catenin accumulation [21]. Considering that phosphorylation of LRP is a hallmark of WNT pathway activation since it disassembles β-catenin destruction complex, Merlin presents as a key antagonist of WNT/ β-catenin signaling. Moreover, Merlin can also directly diminish the availability of free cytoplasmic β-catenin by physically interacting with it and localizing it to the cell membrane, where β-catenin establishes adherens junction together with E-cadherin and α-catenin [25]. Furthermore, phosphorylation of β-catenin at Ser19 and Ser605 by Rac1-activated JNK2 kinase facilitates its nuclear transport. Merlin downregulates Rac1 and weakens JNK2 activation thereby restricting β-catenin’s nuclear transport. Indeed, inhibition of Rac1 decreases WNT activity [26]. Thus, Merlin may control cell proliferation and ensure tissue organization by directly or indirectly controlling β-catenin’s cytoplasmic availability and translocation into the nucleus, consequently blunting WNT/β-catenin signaling (Fig. 1B).

### TGF-β signaling pathway

The TGF-β signaling pathway is involved in the regulation of branching morphogenesis and extracellular matrix deposition such as in the development of the salivary gland [27]. Dysregulated TGF-β signaling is known for causing developmental abnormalities, such as Marfan and Loeys-Dietz syndromes [28]. In cancer biology, TGF-β signaling operates in a context-dependent manner, where it suppresses tumor growth in early stage cancer and promotes disease progression by inducing EMT and metastasis in late stages [29]. Canonical TGF-β signaling leads to the phosphorylation and activation of SMAD2/3 transcription factors by TGF-*β* receptors that, together with co-stimulator SMAD4, associate with transcriptional co-activators or co-repressors to induce or inhibit gene expression, respectively. The TGF-β signaling pathway is blocked by its endogenous inhibitor SMAD7 [29]. Interestingly, SMAD7 levels were decreased concomitantly with loss of Merlin in breast tumor patient samples and cell line, resulting in increased SMAD2/3 phosphorylation and SMAD-driven luciferase activity. The enhancement of TGF-β signaling was involved in increased reliance of *NF2*-deficient breast cancer cells on aerobic glycolysis, a metabolic advantage that sustains cell malignancy [30].

Furthermore, non-canonical TGF- β signaling has been found to be associated with Merlin activity as well. SMAD-independent TGF-β signaling activates PAK2 through the binding of Cdc42 and Rac1. In mesenchymal cells, PAK2 phosphorylates Merlin and inhibits its contact growth inhibition role; this may be one mechanism by which TGF-β signaling promotes growth and contributes to malignancy in late stage cancers. However, in epithelial cells, the binding of Merlin to epithelial-enriched protein Erbin limits the amount of free Merlin to be phosphorylated and inhibited by PAK2. In such context, Merlin’s role in repressing cell overproliferation is maintained. It is likely that Merlin/Erbin disrupts the association between Cdc42/Rac1 and PAK, hinders the latter kinase activation, and imposes a restriction on TGF-β*-*dependent proliferative activity [31]. Moreover, Cho et al. (2018) have reported that *NF2*/Merlin activates non-canonical TGF-β type II receptor (TGFIIR) signaling. TGFIIR can inhibit TGF-β type I receptor (TGFIR) and block its non-canonical oncogenic activity. In conditions of *NF2*/Merlin loss, TGFIIR expression is downregulated, resulting in the activation of TGFIR. As a kinase, TGFIR phosphorylates and inhibits the Raf kinase inhibitor protein (RKIP). Therefore, activated Raf, a downstream effector of Ras signaling, induces ERK activation and causes tumorigenesis through p53 and E-cadherin downregulation and cell cycle upregulation [32]. Overall, Merlin has different approaches to stall TGF-β signaling (Fig. 1C). The paradoxically contrasting effects of TGF-β signaling in cancer may be due to the loss of tumor suppressors, such as Merlin, that normally interfere with TGF-β activation. It is possible that the same scenario occurs in developing tissue, where Merlin deficiency may unbalance TGF-β signaling causing aberrant developmental defects.

### Receptor tyrosine kinase signaling

Receptor tyrosine kinases (RTKs) are transmembrane receptors. In a context-dependent manner, ligands bind to extracellular domain of RTKs leading to dimerization of receptor monomers, phosphorylation of tyrosine residues on the intracellular domain, and activation of downstream pathways that play a role in a variety of cell behaviors, such as cell proliferation, survival, and migration. Unrestrained activation of RTK impairs development and can also cause cancer [33]. The cell membrane availability of the RTKs platelet-derived growth factor receptor (PDGFR) and epidermal growth factor receptor (EGFR), and consequently their downstream signaling, are regulated by Merlin, representing another mechanism by which Merlin keeps check on cell proliferation.

PDGFR/PDGF signaling is involved in various developmental functions, such as proliferation, migration, survival, deposition of extracellular matrix, and tissue remodeling factors [34]. Schwannoma primary human tissue and cell lines lack Merlin expression and were concomitantly found to express high levels of PDGFR and increase activation of its readouts, PI3K/AKT and MAPK/Erk1/2. Conversely, overexpression of *NF2* in HEI 193 schwannoma cell line inhibited PDGFR/PDGF activity. Furthermore, the activation of PI3K/AKT and MAPK/Erk1/2 was also downregulated in cells overexpressing *NF2* [35]. A mechanistic approach of how Merlin negatively modulates PDGFR may be explained by interactions that both proteins have on the inner face of the cell membrane. For instance, the autophosphorylation and consequent activation of PDGFRβ signaling is elevated by its association with the Na+/H+ exchange regulatory cofactor (NHERF). Interestingly, NHERF (also known as ERM binding protein 50 (EBP50)) binds the N-terminal domain of Merlin. As a PDZ domain-containing protein, NHERF/EBP50 plays a role in membrane receptor trafficking to endosomal compartments for lysosomal degradation or recycling back to the membrane [35, 36]. Therefore, it is proposed that Merlin interacts with PDGFRβ via NHERF/EBP50, causing receptor internalization, degradation, and overall downstream signal attenuation.

The ErbB protein family comprises 4 members, ErbB1–4, and is also referred to as human epidermal growth factor receptor (Her). ErbB RTKs are involved in several steps of mammalian development, such as the onset of tissue embryogenesis, cell fate determination, and tissue specialization. Moreover, ErbB is involved in adult tissue maintenance by regulating cell survival and apoptosis [37]. Increased membrane levels, phosphorylation and activity of ErbB2 and ErbB3 are observed in peripheral nerves of *Nf2*-deficient mice and patients with schwannomas, contributing to tumor growth. Cell proliferation was decreased by halting ErbB2 phosphorylation with the EGFR kinase inhibitor Lapatinib [38, 39]. Furthermore, generation of a transgenic, renal tubule-specific *Nf2* knockout mouse resulted in renal invasive carcinoma with elevated EGFR signaling. In vitro proliferation of these renal tumor cells was EGF-dependent and treatment with Erlotinib arrested cell expansion. In addition, re-introduction of *Nf2* also reversed cell proliferation supporting the role of Merlin in controlling EGF/EGFR signaling in the renal system [40]. In *Nf2*-deficient schwannomas, the Hippo transcription factor YAP induces the transcription of prostaglandin and amphiregulin, which activate EGFR [41]. Similar to its regulation of PDGFR availability, Merlin also blocks the internalization and signaling of ErbB RTKs [42]. Merlin is recruited to nascent cell-cell adherens junctions and stabilizes the interaction between cadherin cell adhesion-associated catenins and the cytoskeleton structure. This interaction inhibits the activity of EGFR as the cell senses intercellular forces to stall cell growth [43]. Collectively, the availability of transmembrane PDGFR and EGFR for signaling reveals Merlin’s modulation of cell surface properties in the cell cortex (Fig. 1D).

### Notch signaling pathway

Notch signaling is crucial for cell differentiation, stem cell maintenance, proliferation, migration, and apoptosis. Induction of these cell mechanisms is instrumental for the regulation of developmental processes, such as lateral inhibition, lineage decision, and inductive signaling [4]. In a context dependent manner, Notch ligands in the signal-emitting cells bind to Notch receptors in the signal-receiving cells. The receptors undergo proteolytic cleavage resulting in the release of their receptor intracellular domain (ICD) into the cytoplasm. The ICD translocates to the nucleus and form a transcriptional complex with other DNA-binding proteins to activate target genes, such as HES1 and SOX9 [44].

Deficiency of Notch function in development can manifest as Alagille syndrome, caused mainly by loss-of-function mutation or deletion of Jag1 ligand [45] or CADASIL, resulting from mutation in the Notch 3 receptor, among other disorders [46]. Notch-induced defects can also result from overexpression of either ligands or receptors of the Notch pathway leading to aberrant signaling. This is illustrated by the development of intrahepatic bile ducts where overexpression of Notch 2 receptor, caused by loss of *NF2* and compromised Hippo activity, induced cholangiocyte overproliferation and excessive bile duct development [44]. While gain-of-function mutations in Notch are recurrent in most hematological malignancies, they are not so frequent in breast and adenoid cystic carcinomas [47].

Recognizing Merlin’s modulation of RTKs in the cell membrane, it is likely that Merlin also regulates membrane localization of Notch receptors. This represents a key node of regulation since the membrane availability of Notch receptors determines the rate of Notch signaling. Merlin’s restraint on Notch activity was first proposed in the regulation of imaginal epithelial proliferation and differentiation in *Drosophila.* The physical interaction between Merlin and Expanded, another component of the FERM family of proteins, resulted in the removal of Notch receptors from the cell membrane. Merlin and Expanded might confine Notch transmembrane receptors at locations in the cell membrane marked for endocytosis and degradation by lysosomes. Therefore, loss of Merlin compromises the formation of the complex with Expanded, allowing Notch receptor abundance in the cell membrane and signaling [48] (Fig. 1E). Although not elucidated yet, it may be possible that Merlin also clears Notch ligands from the cell membrane in the signal-emitting cells, further limiting Notch activity.

### Hedgehog signaling pathway

The Hedgehog (Hh) signaling pathway is implicated in the patterning of limbs, internal organs, and body heights in humans [49]. In the absence of secreted Hh ligands, *Desert (Dhh), Indian (Ihh),* and *Sonic (Shh)*, Patched (PTCH) transmembrane protein represses the G-protein coupled receptor Smoothened (SMO). As a result, SMO-dependent signal transduction to GLI transcription factors is compromised. The binding of Hh ligands to PTCH induces GLI to translocate to the nucleus where it activates gene expression [50]. *Shh* ligand activates Hh signaling and promotes proliferation of granule neuron precursor cells in the developing cerebellum. However, dysregulated stimulation by *Shh* leads to uncontrolled cell division resulting in medulloblastoma, the most common type of brain tumor in children [51]. Details about a possible regulation of Hh signaling by Merlin are yet to emerge. However, in *NF2/*Merlin-deficient breast cancer cells, reactive oxygen species (ROS) accumulation resulting from decreased levels of anti-oxidant, GSH, lead to elevated Hh signaling [52]. ROS are known to induce conformational changes in proteins, especially by oxidizing thiol groups on cysteine residues, and affect their enzymatic activity, post-translational modifications, or protein-protein interaction [53]. Therefore, it is possible that ROS oxidize and inhibit PTCH, enabling the release of SMO from PTCH repression. Alternatively, ROS may also modify GLI’s ability to enable ligand-independent activation. It is also possible that ROS may upregulate expression of Hh ligands or enable nuclear translocation of GLI proteins. Overall, Merlin likely restricts GLI activity by modulating the oxidative intracellular milieu (Fig. 1F). Given this pathway’s important role in development patterning, it is likely that redox-imbalance-driven Hh activation may also be prevalent in tumors with loss of *NF2* function*.*

## Conclusion

**Fig. 2:**
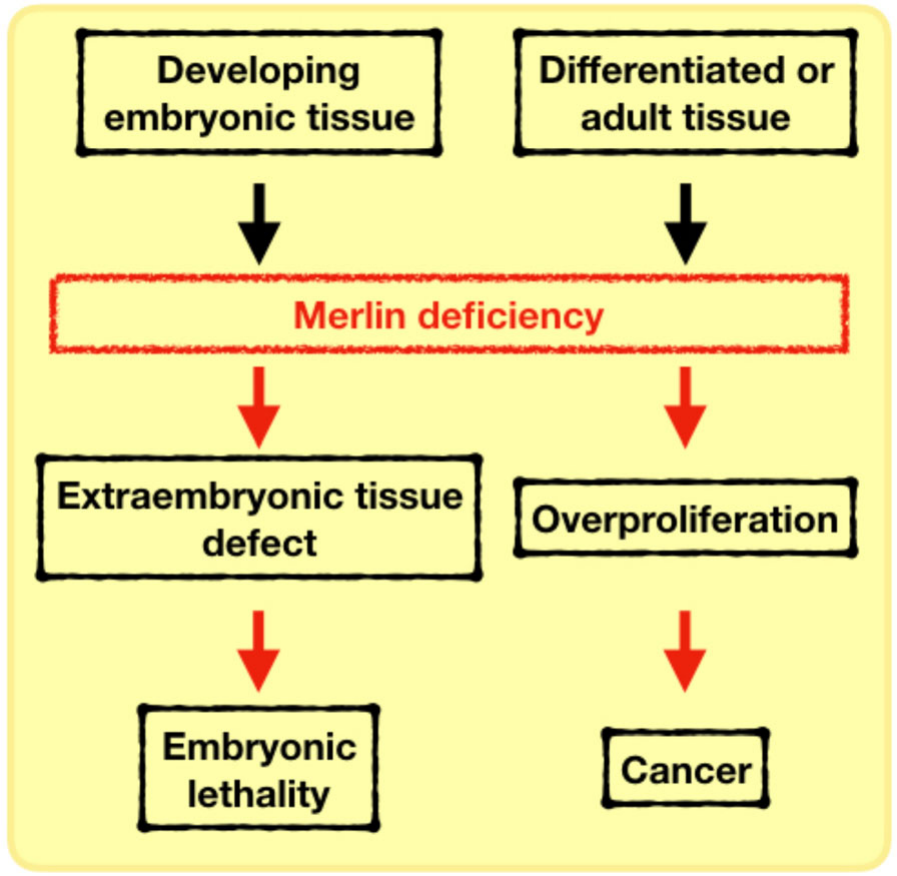
Merlin depletion negatively impacts embryonic development and tissue maintenance. Merlin is indispensable for normal extraembryonic structure formation and its loss is embryonic lethal. In differentiated and normal tissues, Merlin loss results in cell overproliferation due to defective contact-growth inhibition and promotes cancer

Merlin loss in early stages of development is lethal. Loss of Merlin’s function in differentiated tissue results in aberrant cell proliferation causing benign and malignant tumor formation and progression (Fig. 2). These outcomes are explained by the fact that several of the signaling pathways with which Merlin intersects are instrumental in keeping optimally active development programs and adult tissue maintenance. Therefore, Merlin represents an indispensable key regulator of signals in different phases of an organism’s development, ranging from an embryo to a fully differentiated, functional system. Shedding light on how Merlin acts in different contexts is undoubtedly necessary for a comprehensive understanding of its loss in impacting disease onset and development. There is still no strategy to directly overcome Merlin deficiency in Neurofibromatosis type 2 and cancers; therefore, targeting the collateral effect of loss of Merlin function seems like a promising approach. Targeting aberrantly activated developmental signaling in Merlin-deficient malignancies offers a new therapeutic direction with several inhibitors having already received FDA approval for targeting these pathways (e.g. Hippo, WNT/β-catenin, TGF-β, RTK, Notch, and Hedgehog). In addition, investigation of developmental syndromes from the perspective of Merlin may evolve the understanding of disorders and consequently therapeutic strategy as well.

## Data Availability

Not applicable.

## Abbreviations

APC: Adenomatous polyposis coli
CADASIL: Cerebral autosomal dominant arteriopathy with subcortical infarcts and leukoencephalopathy
CK1α: Casein kinase 1α
DCAF: DDB1-and-Cullin 4-associated Factor 1
Dhh: Desert hedgehog
DLL: Delta-like
EBP50: ERM binding protein 50
EGFR: Epidermal growth factor receptor
ERM: Ezrin/Radixin/Moesin
FERM: 4.1, ERM
FZD: Frizzled
GSH: Glutathione
Hh: Hedgehog
ICD: Intracellular domain
Ihh: India hedgehog
JNK2: c-Jun N-terminal kinase 2
LATS: Large tumor suppressor kinase
LRP: Lipoprotein receptor-related protein
MST: Mammalian Sterile20-like kinase
MYPT1: Myosin phosphatase target subunit 1
NF2: Neurofibromin 2
NHERF: Na + − H+ exchange regulatory cofactor
PAK: p21-activated kinase
PDGFR: Platelet-derived growth factor receptor
PKA: Protein kinase A
PTCH: Patched
RBPJ: Recombining binding protein suppressor of hairless
ROS: Reactive oxygen species
RTK: Receptor tyrosine kinase
SH2: Src Homology 2
Shh: Sonic hedgehog
SMO: Smoothened
TCF/LEF: T-cell factor/ lymphoid enhancing factor
TGFR: TGF-β receptor
YAP: Yes-associated protein
